# The Potential of Nanobody-Targeted Photodynamic Therapy to Trigger Immune Responses

**DOI:** 10.3390/cancers12040978

**Published:** 2020-04-15

**Authors:** Irati Beltrán Hernández, Mathieu L. Angelier, Tommaso Del Buono D’Ondes, Alessia Di Maggio, Yingxin Yu, Sabrina Oliveira

**Affiliations:** 1 Pharmaceutics, Department of Pharmaceutical Sciences, Faculty of Science, Utrecht University, 3584 CG Utrecht, The Netherlands; i.beltranhernandez@uu.nl (I.B.H.); t.delbuonodondes@students.uu.nl (T.D.B.D.); 2 Cell Biology, Neurobiology and Biophysics, Department of Biology, Faculty of Science, Utrecht University, 3584 CH Utrecht, The Netherlands; m.l.angelier@students.uu.nl (M.L.A.); a.dimaggio@students.uu.nl (A.D.M.); Y.Yu@uu.nl (Y.Y.)

**Keywords:** targeted photodynamic therapy, nanobodies, EGFR, DAMPs, immune stimulation

## Abstract

Nanobody-targeted photodynamic therapy (NB-PDT) has been recently developed as a more tumor-selective approach rather than conventional photodynamic therapy (PDT). NB-PDT uses nanobodies that bind to tumor cells with high affinity, to selectively deliver a photosensitizer, i.e., a chemical which becomes cytotoxic when excited with light of a particular wavelength. Conventional PDT has been reported to be able to induce immunogenic cell death, characterized by the exposure/release of damage-associated molecular patterns (DAMPs) from dying cells, which can lead to antitumor immunity. We explored this aspect in the context of NB-PDT, targeting the epidermal growth factor receptor (EGFR), using high and moderate EGFR-expressing cells. Here we report that, after NB-PDT, the cytoplasmic DAMP HSP70 was detected on the cell membrane of tumor cells and the nuclear DAMP HMGB1 was found in the cell cytoplasm. Furthermore, it was shown that NB-PDT induced the release of the DAMPs HSP70 and ATP, as well as the pro- inflammatory cytokines IL- 1β and IL-6. Conditioned medium from high EGFR-expressing tumor cells treated with NB-PDT led to the maturation of human dendritic cells, as indicated by the upregulation of CD86 and MHC II on their cell surface, and the increased release of IL-12p40 and IL-1β. Subsequently, these dendritic cells induced CD4+ T cell proliferation, accompanied by IFNγ release. Altogether, the initial steps reported here point towards the potential of NB-PDT to stimulate the immune system, thus giving this selective-local therapy a systemic reach.

## 1. Introduction

Photodynamic therapy (PDT) was first approved in 1993 for the treatment of bladder cancer and, since then, its use has expanded to many other oncological indications, such as lung, brain, esophagus, skin, and head and neck cancer [1]. This treatment relies on the action of a photosensitizer (PS), i.e., a light activatable compound that accumulates into cells and becomes cytotoxic when excited with light of a particular wavelength. Upon PS activation, reactive oxygen species are generated that ultimately lead to cell death [1,2]. Besides direct cytotoxicity, damage to the tumor vasculature and the potential to stimulate an antitumor immune response have been reported [2,3,4]. This last aspect is of particular interest since a local treatment such as PDT, making use of locally applied light at the tumor site, could develop systemic effects via the activation of the immune system.

Despite being a selective and controllable treatment against tumor cells, therapies known to be considerably aggressive towards all tissues (i.e., chemo- and radiotherapy) are still the mainstream clinical practice, when it comes to combat cancer [5]. Some current barriers for the establishment of PDT in routine practice are the required technology, complex dosimetry, limited tissue penetration of light and/or PS, lack of tumor specificity and prolonged skin photosensitivity [1,5]. Efforts have been put into improving the selectivity of PDT towards the cancer cells by using antibodies, an approach which is now in phase II clinical trial (NCT02422979) [6]. Conjugation of a water-soluble PS (IRDye700DX) to an antibody allows the PS to be specifically delivered to cancer cells overexpressing a certain antigen on the cell membrane. In this manner, two levels of specificity are established: the delivery of PS to target-expressing cells and the local application of light at the tumor site. 

Another strategy to render PDT more tumor-selective is nanobody-targeted PDT (NB-PDT), which also utilizes the near-infrared PS IRDye700DX. In this case, however, a nanobody is used to provide the first level of specificity [7,8]. NBs are the smallest binding domains found in nature (~15 kDa) and consist uniquely of the variable domain of heavy chain antibodies present in Camelids [9]. The use of a NB for PDT brings a series of advantages over antibody-targeted PDT. Their small size enables rapid tumor accumulation, with a homogeneous distribution, and a rapid clearance from circulation when unbound [9,10,11]. In the context of PDT, this allows the application of the light shortly after conjugate administration, ensures more extensive tumor damage, and reduces the chances of phototoxicity present in both PDT and antibody-targeted PDT protocols. In vitro, NB-PDT has been reported to be a very specific, selective, and potent approach to kill cancer cells expressing a variety of membrane receptors, such as epidermal growth factor receptor (EGFR) [7,8], human epidermal growth factor receptor 2 (HER2) [12], hepatocyte growth factor receptor (c-Met) [13], and G protein- coupled receptor (GPCR) [14]. Furthermore, NB-PDT has proven very effective in inducing tumor necrosis in an orthotopic mouse model of head and neck cancer expressing EGFR [7], and to induce significant tumor regression in an orthotopic mouse model of breast cancer expressing HER2 [12]. Additionally, similar to the observations with conventional PDT, damage to the tumor vasculature induced by NB-PDT has also been reported [15]. 

Subsequently, we aimed to explore the third component of responses triggered by PDT, i.e., whether NB-PDT can also trigger the immune system, possibly leading to an antitumor response, which would greatly add to the potential that NB-PDT has shown so far. This ability of PDT to stimulate immune cells has been linked to the fact that PDT is able to induce an immunogenic cell death (ICD). This type of cell death is accompanied by the release and exposure of several molecules from the dying cells, such as endogenous cytokines and damage-associated molecular patterns (DAMPs), which can be recognized by various activating receptors (e.g., pattern recognition receptors) on immune cells and, thereby, stimulate subsequent immune responses [16]. For instance, heat shock protein 70 (HSP70), high mobility group box 1 protein (HMGB1), calreticulin, and ATP can all act as DAMPs described to be exposed/released after PDT and associated with activation of innate immunity. These can trigger the local innate immune system (e.g., dendritic cells, DCs), thus paving the way towards the development of an adaptive memory immune response [3,17], which is indispensable for preventing tumor recurrence and the formation of tumor metastasis in the long term. Importantly, the successful development of an anti-tumor response post-PDT relies largely on the initial induction of ICD [18].

In this study, we address the immunogenic potential of NB-PDT for the first time, from the initially induced tumor cell death mechanism, to the last step of the immune reaction involving activation of T cells. The EGFR-targeting NB-PS conjugate named 7D12-PS was employed for NB-PDT, a conjugate that has already shown its potency for the selective killing of EGFR-expressing cells [8]. Due to its slow internalization, after binding to EGFR, 7D12-PS is mostly present on the cell membrane when light is applied, thereby inducing membrane damage and subsequent cell death [8]. In vivo, this nanobody has been described to present rapid tumor accumulation and homogenous distribution [10,11] and, upon illumination, induces extensive tumor damage, as well as vascular effects [7,15]. In order to take into account possible inter-tumor heterogeneity of target expression, two different tumor cell lines were included: the A431 cell line that overexpresses EGFR and the scc- U8 cell line expressing moderate EGFR levels, which more closely resemble the in vivo levels [19,20]. With this in vivo mirroring in mind, a highly cytotoxic NB-PDT (LD100) and a mild treatment (LD50) are also compared here, since uneven light penetration and tumor heterogeneity would likely lead to different degrees of cytotoxicity in vivo. Our results show that NB-PDT induces rapid necrosis accompanied by exposure/release of major DAMPs and cytokines from the dying cells. This release of immunogenic factors leads to the maturation of DCs which subsequently activate CD4+ T cells, thereby demonstrating the immunogenicity triggered by NB-PDT.

## 2. Results

### 2.1. Necrosis Is the Main Cell Death Mechanism Induced by NB-PDT

**Figure 1:**
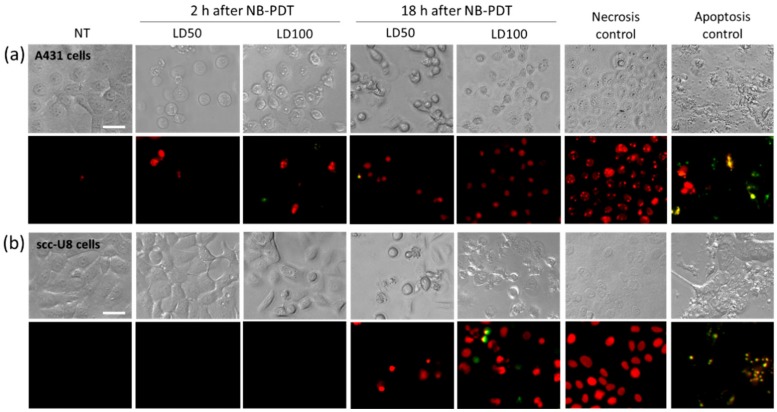
Cell death mechanism induced by nanobody-targeted photodynamic therapy (NB-PDT). Tumor cells were left untreated (NT) or treated with NB-PDT using 7D12-PS (LD50 or LD100) and stained with propidium iodide (PI) for necrotic cells (red) and caspase 3/7 for apoptotic cells (green), controls for necrosis and apoptosis were included. Microscopy images of (**a**), A431 cells and (**b**), scc-U8 cells were taken 2 and 18 h after NB-PDT. Top panels depict the transmitted light image and bottom panels the merged images of necrotic and apoptotic cells. Scale bar, 20 µm.

Taking into account possible variations in EGFR expression and in light penetration, the type of cell death induced by NB-PDT was investigated after mild (LD50) and highly cytotoxic (LD100) NB-PDT, on high and moderate EGFR-expressing cells, i.e., A431 and scc-U8 cells, respectively. An overview of the LD50 and LD100 used in this study for each cell line is depicted on Figure S1. The cell death mechanism was studied 2 and 18 h after treatment, by means of fluorescent dyes denoting apoptosis or necrosis. Necrosis was the main cell death mechanism triggered by NB-PDT on A431 cells (Figure 1a). Cell morphology resembled the positive control for necrosis, reflecting the extensive damage that this conjugate induces on the cell membrane. Morphological changes on the cells 2 h after treatment, i.e., rounded up cells, indicate that cells are rapidly affected by NB-PDT. Contrary to A431 cells, scc-U8 cells showed only slight morphological changes shortly after treatment, while after 18 h a mixture of both necrotic and apoptotic cells could be detected under the highly cytotoxic condition (Figure 1b). No cell death signal was detected upon the use of the individual components of NB-PDT, indicating the lack of toxicity when using the conjugate or light alone (Figure S2a). 

### 2.2. Cellular Localization of HSP70 and HMGB1 Change on Tumor Cells Treated with NB-PDT

**Figure 2:**
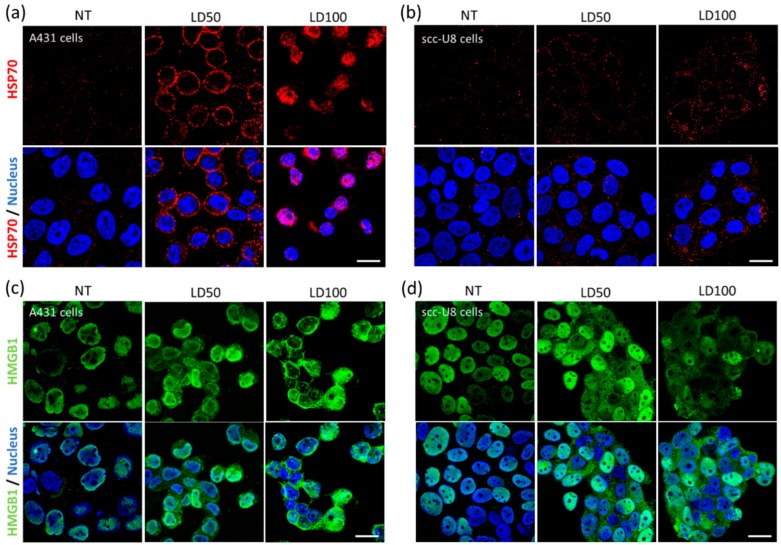
Cellular localization of heat shock protein 70 (HSP70) and high mobility group box 1 protein (HMGB1) on tumor cells treated with NB-PDT. Tumor cells were left untreated (NT) or treated with NB-PDT using 7D12-PS (LD50 or LD100), and 4 h later stained for HSP70 or HMGB1. Staining of HSP70 (red) was performed on non-permeabilized (**a**), A431 cells and (**b**), scc-U8 cells. Intracellular staining of HMGB1 (green) was performed on (**c**), A431 cells and (**d**), scc-U8 cells. Cell nuclei were additionally stained with DAPI (blue). Top panels depict only the damage-associated molecular pattern (DAMP) signal, while merged images are shown on the bottom panels. Scale bar, 20 µm.

Changes in the localization of several DAMPs within tumor cells were investigated 4 h after treatment with NB-PDT. This early time point was chosen to allow visualization of not yet severely damaged cells and facilitate detection of these DAMPs in the different cell compartments. Here, the DAMPs HSP70 (a cytoplasmic chaperone) and HMGB1 (a nuclear chromosomal protein) were considered. An increase of HSP70 on the cell membrane was observed after mild NB-PDT in comparison to untreated cells (Figure 2a,b) and controls of only light or conjugate (Figure S2b). On the other hand, HSP70 was detected in the cytoplasm and nucleus of cells after the highly cytotoxic treatment in the case of A431 cells. This is in line with the extensive damage imposed on the cell membrane with this NB-PDT condition, resulting in a permeabilized membrane allowing intracellular detection of HSP70. Overall, the membrane staining of HSP70 was more evident on A431 cells than on scc-U8 cells and no extensive membrane damage was yet observed on scc-U8 cells after the highly cytotoxic treatment in the time frame of this experiment. 

HMGB1 was predominantly present in the nucleus of untreated A431 and scc-U8 cells (Figure 2c,d), as well as control cells exposed to only light or conjugate (Figure S2c). After mild and highly cytotoxic NB-PDT, HMGB1 was detected in both nucleus and cytoplasm of the cells. Of note, HMGB1 was found to be almost completely excluded from the nucleus in a large number of A431 cells under the highly cytotoxic condition, again indicative of the substantial damage inflicted by NB-PDT to this cell line at early time points. Overall, both A431 and scc-U8 cells depicted a similar behavior regarding the changes in localization of both DAMPs.

### 2.3. NB-PDT Induces ATP and HSP70 Release from Treated Tumor Cells

**Figure 3:**
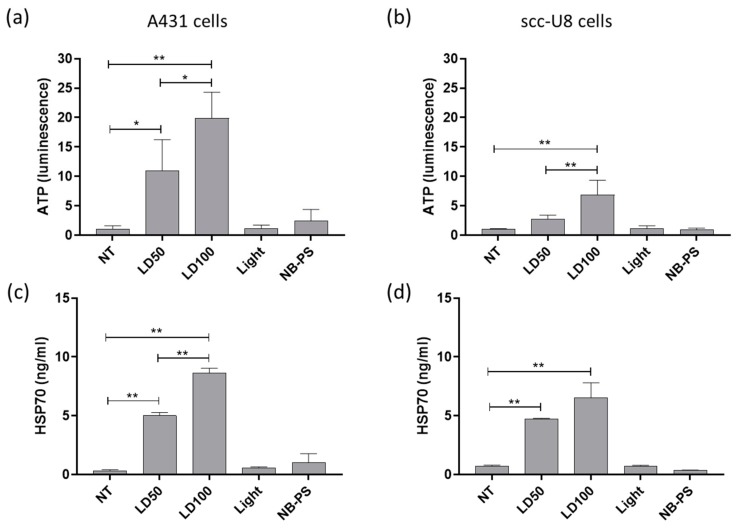
Quantification of ATP and HSP70 release from tumor cells after NB-PDT. ATP in the supernatant was detected 4 h after NB-PDT via a luminescence assay and graphs show luminescence values relative to untreated cells for (**a**), A431 cells and (**b**), scc-U8 cells. Additionally, released HSP70 was detected 24 h after treatment using ELISA on (**c**), A431 cells and (**d**), scc-U8 cells. NT, untreated; LD50, mild cytotoxic NB-PDT; LD100, highly cytotoxic NB-PDT; Light, only light control; NB-PS, only nanobody-photosensitizer (NB-PS) conjugate control. Significance is displayed as * *p* ≤ 0.05 and ** *p* ≤ 0.01.

For both cell lines, a higher amount of ATP was detected in the supernatant of tumor cells 4 h after NB-PDT, under both mild and highly cytotoxic conditions (Figure 3a,b). A431 cells treated with NB-PDT showed higher release of ATP when compared with scc-U8 cells. Similarly, high concentrations of HSP70 were detected in the supernatant of treated tumor cells (Figure 3c,d). Nonetheless, in this case, the HSP70 concentration in the supernatants was comparable between cell lines. For both DAMPs, release was always more pronounced when cells were treated with the highly cytotoxic NB-PDT.

### 2.4. Cytokine Levels Released by Tumor Cells Are Altered after NB-PDT

**Figure 4:**
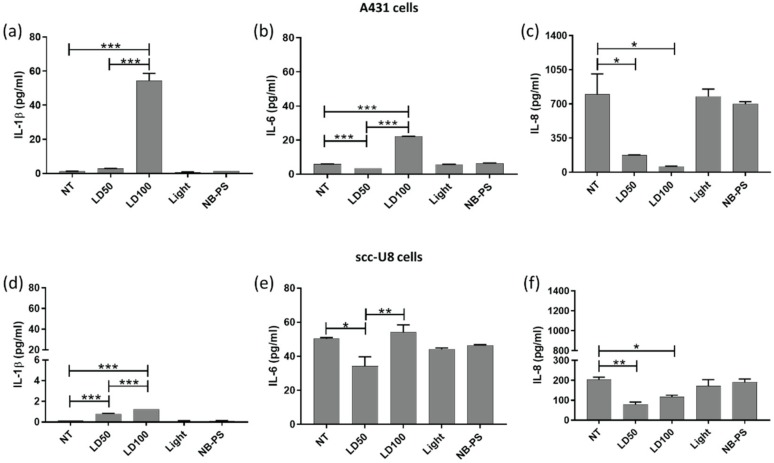
Quantification of IL-1β, IL-6 and IL-8 release by tumor cells treated with NB-PDT. A431 or scc-U8 cells were treated with NB-PDT and the concentration of several cytokines in the supernatant was quantified 24 h later. Graphs display the quantification of IL-1β, IL-6 and IL-8 on A431 cells (**a**, **b**, and **c**, respectively) and on scc-U8 cells (**d**, **e**, and **f**, respectively). NT, untreated; LD50, mild cytotoxic NB-PDT; LD100, highly cytotoxic NB-PDT; Light, only light control; NB-PS, only NB-PS conjugate control. Significance is displayed as * *p* ≤ 0.05, ** *p* ≤ 0.01 and *** *p* ≤ 0.001.

Release of particular cytokines from tumor cells was investigated after NB-PDT. High concentrations of the proinflammatory cytokines IL-1β (Figure 4a) and IL-6 (Figure 4b) were quantified in the supernatants of A431 cells treated with the highly cytotoxic NB-PDT. Changes regarding the levels of these cytokines were less pronounced on the moderate-EGFR expressing scc- U8 cells (Figure 4d,e), but similar trends were detected. Furthermore, both tumor cell lines secreted considerable amounts of IL-8, which were substantially reduced after both mild and highly cytotoxic NB-PDT (Figure 4c,f).

### 2.5. Maturation of Dendritic Cells Is Induced by NB-PDT Treated Tumor Supernatants

**Figure 5:**
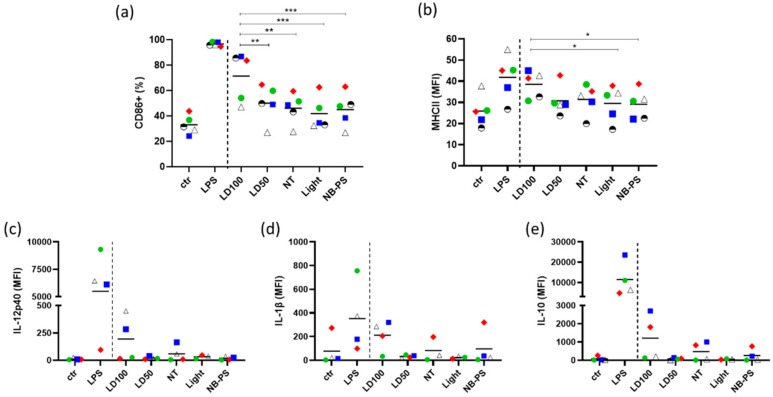
Phenotypic maturation and cytokine release of monocyte-derived dendritic cells (moDCs) incubated with supernatant of NB-PDT treated tumor cells. A431 cells were treated with NB-PDT, the supernatant was collected 24 h later and incubated with immature moDCs for another 24 h. Surface marker expression on moDCs was measured with flow cytometry, and cytokine release was assessed by Luminex. (**a**), Percentage of CD86 positive moDCs. (**b**), Median fluorescence intensity (MFI) corresponding to MHCII surface expression on moDCs. Each moDC donor (n = 5) is represented by a different symbol and color. ctr, unstimulated DCs; lipopolysaccharide (LPS), LPS-stimulated DCs; NT, untreated tumor cells; LD50, mild cytotoxic NB-PDT; LD100, highly cytotoxic NB-PDT; Light, only light control; NB-PS, only NB-PS conjugate control. Significance is displayed as * *p* ≤ 0.05, ** *p* ≤ 0.01, and *** *p* ≤ 0.001. C-E, MFI corresponding to the release by moDCs of (**c**), IL-12p40; (**d**), IL-1β; and (**e**), IL-10 (n = 4). No statistical significance was found between groups due to the intrinsic differences between donors.

Monocyte-derived DCs (moDCs) were incubated with the conditioned medium of tumor cells treated with NB-PDT and the expression of two maturation markers, MHCII (an antigen presenting molecule) and CD86 (a costimulatory molecule), on the surface of moDCs was evaluated. Lipopolysaccharide (LPS) stimulation was used as a positive control. Subsequently, increase of the CD86+ population was detected only when moDCs were incubated with LPS or conditioned medium of cells treated with highly cytotoxic NB-PDT (Figure 5a,b). All the other groups, including mild NB-PDT and controls of the single components of the treatment, failed to induce significant upregulation of this maturation marker. The same trend was observed for the upregulation of MHCII on moDCs, although significance was affected by the intrinsic differences between donors. 

Besides phenotypic maturation, further activation of moDCs was investigated by measuring their release of IL-12, IL-1β, and IL-10 after incubation with NB-PDT treated tumor supernatant. First, IL12-p70 detection in the supernatant of moDCs was attempted since this is the prime cytokine released by DCs to activate T cells. Nonetheless, results were negative due to the detection limit of the assay employed (not shown). On the other hand, a trend towards increased release of other main cytokines, i.e., IL-12p40 and IL-1β, was detected in moDCs incubated with tumor supernatant from the highly cytotoxic condition (Figure 5c,d), which can be associated with an immunostimulatory profile. Release of IL-10, an anti-inflammatory cytokine, was also found to be elevated in these moDCs (Figure 5e). From the five different donors from which moDCs were isolated, only the moDCs of one of these donors (green circles in Figure 5) did not respond to NB-PDT in terms of phenotype maturation and cytokine release. 

### 2.6. Dendritic Cells Exhibit a Stronger Ability to Induce CD4+ T Cell Activation after Stimulation with NB-PDT Treated Tumor Supernatants

**Figure 6:**
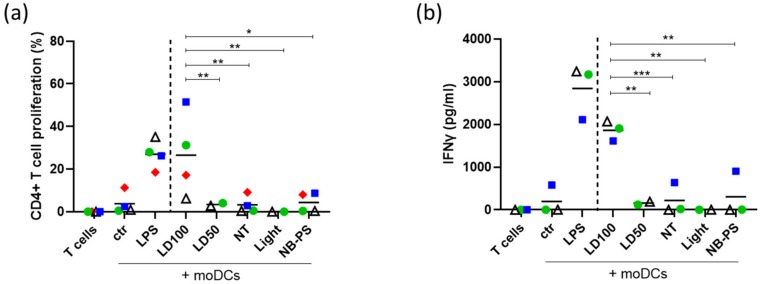
Enhanced proliferation and IFNγ release of CD4+ T cells induced by moDCs stimulated with supernatant of NB-PDT treated tumor cells. A431 cells were treated with NB-PDT, the supernatant was collected 24 h later and incubated with immature moDCs for another 24 h. moDCs were then co- incubated with allogeneic CFSE-labeled CD4+ T cells in a 1:10 ratio. After 6 days, CD4+ T cell proliferation was measured with flow cytometry and IFNγ release was assessed by ELISA. (**a**), Percentage of CD4+ T cells with weak CFSE signal, thus proliferating cells (n = 4). (**b**), Quantification of released IFNγ by CD4+ T cells (n = 3). Each combination of allogeneic donors is represented by a different symbol and color. ctr, unstimulated DCs; LPS, LPS-stimulated DCs; NT, untreated tumor cells; LD50, mild cytotoxic NB-PDT; LD100, highly cytotoxic NB-PDT; Light, only light control; NB-PS, only NB-PS conjugate control. Significance is displayed as * *p* ≤ 0.05, ** *p* ≤ 0.01 and *** *p* ≤ 0.001.

After detecting increased expression of MHCII and CD86 on the surface of moDCs, we speculated that these stimulated moDCs, upon crosslinking, would increase CD4+ T cell activation. To prove this, stimulated moDCs were cocultured with allogeneic naïve CD4+ T cells and, after a 6- day coculture, the activation of T cell was investigated, which involves T cell proliferation accompanied by IFNγ production. In this context, moDCs stimulated with supernatant of tumor cells treated with highly cytotoxic NB-PDT were able to enhance the proliferation (Figure 6a) and IFNγ production (Figure 6b) of CD4+ T cells, compared to untreated moDCs. LPS treated moDCs served as positive control. On the contrary, no T cell activation was detected for any other NB-PDT condition or control.

## 3. Discussion

NB-PDT has been developed as a more tumor-selective alternative to PDT and has already shown great potential in vitro and in preclinical models [7,8,13,14]. NB-PDT allows light application shortly after PS administration, ensures extensive tumor damage, and is expected to reduce the post- treatment phototoxicity associated with some PDT protocols. Both NB-PDT and its conventional counterpart initially cause extensive damage to the primary tumor and to the tumor vasculature [2,15]. In addition, the conventional approach has been described to also stimulate an antitumor immunity [3,4]. Consequently, this local treatment can induce a systemic effect, a feature that is highly desirable for protection against metastasis and recurrences. In this study, for the first time, the immunomodulatory potential of NB-PDT is addressed. We describe the first steps regarding this aspect: in vitro NB-PDT induces a large extent of necrosis, leads to changes in cellular localization and release of well-known DAMPs and proinflammatory cytokines from tumor cells, and ultimately induces the maturation of moDCs and subsequent CD4+ T cell activation.

It has been firmly established that PDT can be a potent inducer of ICD, which together with the triggered initial local inflammation, can promote the development of an adaptive immune response against the tumor [16]. Nonetheless, the development of such a response is not always successful since it is ultimately influenced by the type of PS and its cellular localization, tumor type and stage, and illumination protocol, among other factors [21]. To cover some of these aspects, we employed: (1) two tumor cell lines expressing high (A431 cells) and moderate (scc-U8 cells) EGFR levels, and (2) two concentrations of the conjugate 7D12-PS to yield mild (LD50) and highly cytotoxic (LD100) NB-PDT. In our previous studies, an additional NB-PS was employed, i.e., 7D12-9G8-PS. This nanobody has been described to lead to faster internalization of EGFR [22] and consequently to a more pronounced delivery of PS intracellularly [8], altogether leading to more toxicity in vitro (lower LD50 than 7D12-PS). However, in the context of this study, no significant differences were observed compared to 7D12-PS (Figure S4). Together with the fact that the monovalent nanobody format is expected to distribute more homogenously than a bivalent format [11], this study was focused on one conjugate only, i.e., 7D12-PS. 

Conventional PDT mostly uses hydrophobic PSs which are internalized by the cells and mainly end up in the mitochondria, endoplasmic reticulum, or lysosomes. Consequently, the main cell death mechanism triggered by most PSs is apoptosis due to initial damage to these organelles [5]. We showed that, in the case of NB-PDT using 7D12-PS, necrosis is the main cause of cell death (Figure 1). This is because the conjugate is mostly present on the cell membrane at the time of the light application, as previously reported in [8], leading to its substantial damage. As a result of the loss of plasma membrane integrity, ATP depletion can rapidly occur, which directs the death program towards necrosis [23,24]. Likewise, extensive cell membrane damage has been reported for EGFR- targeted PDT using antibodies [25]. On the other hand, some apoptotic scc-U8 cells were observed when treated with 7D12-PS, pointing towards the idea that lower membrane EGFR levels equals less abrupt damage on the cell membrane, thus giving the cell time to respond in other ways (e.g., apoptosis). Interestingly, the use of 7D12-9G8-PS, which internalizes faster, led to cell death in a more rapid fashion than the monomeric counterpart (Figure S3a), which might be explained by a combination of higher avidity and internalization capacity. Besides necrosis, some degree of apoptosis was triggered by this conjugate, suggesting a similar organelle damage to that of conventional PSs when the NB-PS is internalized. Despite these differences in the induced mechanism of cell death, this did not seem to have a significant effect on the subsequent release of DAMPs (Figure S3b,c). 

Overall, it seems that for NB-PDT the amount of NB-PS conjugate on the membrane is decisive for the induction of rapid necrosis, and we showed this can vary depending on target expression, conjugate concentration or internalizing rate of the conjugate. There is so far not a consensus on whether PDT-induced necrosis or apoptosis is more suitable to trigger an antitumor immune response, although some advocate the latter [26]. Nevertheless, it is likely that in the clinical setting both types of cell death will simultaneously occur, and both can contribute to the successful activation of immune cells due to the release and exposure of DAMPs and other immunogenic molecules from the dying cells [27].

This exposure of immunogenic molecules after NB-PDT was the next event explored in this study. Conventional PDT is described to induce exposure of HSP70, among other chaperones, on the membrane of tumor cells [28,29,30,31]. Membrane exposure of HSP70 was indeed observed on A431 and scc-U8 cells treated with mild NB-PDT, although it was more evident on the high EGFR-expressing cells at the early timepoint studied (Figure 2a,b). When HSP70 is located on the cell membrane, it displays unique functions since it can be recognized by antigen presenting cells and it aids in the cross-presentation of tumor antigens [32]. Hence, mild NB-PDT seems to provide a setting where these mechanisms can take place, while the highly cytotoxic condition relies mostly on a substantial release of DAMPs. Another DAMP, HMGB1, has been described to change its cellular localization after conventional PDT, from nuclear to cytoplasmic, being subsequently released [28,30,33]. The same changes in the localization of HMGB1 were observed here on both A431 and scc-U8 cells treated with NB-PDT (Figure 2c,d). In our case, the highly cytotoxic condition not only induced translocation of this DAMP to the cytoplasm, but it was also excluded from the nucleus in some A431 cells, as also described by other groups using conventional PDT [33].

The release from tumor cells of two DAMPS (i.e., HSP70 and ATP) was also addressed, knowing that both can determine the immunogenicity of the dying cells [27] and their release has been described after conventional PDT [28,29,30,34]. HSP70 and ATP were indeed released from both A431 cells (treated with either 7D12-PS or 7D12-9G8-PS) and scc-U8 cells (treated with 7D12-PS) (Figure 3 and Figure S3b,c). In agreement with the extent of induced cell death, the highly cytotoxic NB-PDT always led to the largest release of these DAMPs, followed by the mild treatment, suggesting passive release of DAMPs from the necrotic cells. Although differences were observed in confocal images taken from A431 and scc-U8 cells (Figure 2a,b), the HSP70 concentration in the supernatants was comparable between cell lines. This can be explained by the fact that HSP70 release was quantified 24 h after treatment, when we expect complete effect of the LD50 and LD100 treatment and no differences between cell lines due to the less extensive damage on scc-U8 early after NB-PDT. Concerning the biparatopic conjugate, this did not yield higher amounts of released ATP than 7D12- PS (measured 4 h after NB-PDT), while the extent of cell death is considerably larger, shortly after treatment. This can be related to the induction of a mix of apoptosis and necrosis by 7D12- 9G8- PS, where ATP is being utilized to execute the programmed cell death mechanism instead of being released. The release of HMGB1 was not investigated in our study, but its change in cellular localization (Figure 2c,d) suggests its release will eventually take place, where it can regulate antigen processing and presentation by DCs [32]. Importantly, one of the hallmarks of ICD, together with HMGB1 and ATP release, is calreticulin surface exposure. Our attempts to explore this event under the microscope have thus far not been successful. Antibody-targeted PDT induces only minimal calreticulin exposure compared to HSP70 or HSP90, as measured by flow cytometry, but is still able to trigger potent ICD [25]. Flow cytometry is a more sensitive technique than fluorescence microscopy, able to detect these slight changes. We believe the use of more sensitive and quantitative techniques would further support the potential of NB-PDT to induce ICD.

Besides DAMPs, cytokines can also be released by dying tumor cells. In this study, we were able to detect an increased release of the proinflammatory cytokines IL-1β and IL-6 from tumor cells treated with the highly cytotoxic NB-PDT (Figure 4). At the same time, IL-8 secretion was considerably decreased after NB-PDT. In this case, IL-8 might be having a protumoral function on the tumor cells, as suggested in [35], since high basal levels of released IL-8 are detected from untreated cells. Others have described modulation of cytokine production of IL-1α, IL-1β, IL-2, IL-4, IL-6, IL-8, IL-10, IL-17A, TNF-α, and IFN-γ on tumor cells treated with conventional PDT [36,37,38], but their increase or decrease seems to highly depend on the cell line and PDT protocol used. Of note, we observed a small but significant decrease of IL-6 release only after mild NB-PDT, which, incidentally, has also been reported after a sublethal PDT dose [36]. This was an unexpected observation and further study will help elucidate this aspect. On the whole, our results point towards an immunostimulatory cytokine profile of the NB-PDT treated tumor cells.

The key element of ICD is the stimulation of immune cells after the release/exposure of immunogenic molecules from dying cells. In this context, DCs are of particular interest due to their ability to bridge innate and adaptive immune response. We have confirmed that indeed moDCs show maturation features when in the presence of supernatant of tumor cells treated with the highly cytotoxic NB-PDT, including upregulation of the maturation markers CD86 and MHC II (Figure 5a,b) and increased secretion of IL12-p40, IL-1β, and IL-10 (Figure 5c,d). It is important to mention that there exist considerable intrinsic differences between donors, which impacts the statistical significance of this set of experiments. Interestingly, we have observed a similar upregulation pattern of maturation markers on moDCs (although statistically nonsignificant) when using conditioned medium of treated scc-U8 cells in a small scale experiment (Figure S4), suggesting a more moderate damage to the tumor cells may lead as well to phenotypic maturation of moDCs. Nevertheless, conclusions should be carefully drawn from such a small scale experiment. Others have reported maturation of DCs induced by conventional PDT, including upregulation of CD40, CD80, CD83, CD86, and MHC II, accompanied by increased secretion of IL-1β, IL-12, nitric oxide, and IFN-γ [26,30,31,34,39]. Although secretion of IL-10 (an immunosuppressive cytokine) has been reported as reduced or absent in some studies [26,34], others like ours show low levels of this cytokine are still secreted by PDT-stimulated DCs [31]. In our case, this might be explained by the fact that the NB-PDT treated tumor supernatants present TLR4 agonist activity (Figure S5), in agreement with the increase of HSP70 and HMGB1 that are TLR4 agonists themselves and can trigger IL-10 release [27].

A crucial event for antitumor immunity is the subsequent activation of naïve T cells by mature DCs. We describe that moDCs stimulated by tumor supernatant of the highly cytotoxic NB-PDT condition are able to provide the adequate stimulatory signals for the proliferation of CD4+ T cells (Figure 6a) and differentiation towards Th1 CD4+ effector T cells, as indicated by the substantial release of IFNγ (Figure 6b). Th1 CD4+ effector T cells are known to have a clear antitumor role and, via IFNγ release, can sustain a potent cellular immune response involving CD8+ cytotoxic T cells [40,41]. Although the activation of these cytotoxic T cells was not investigated in our study, their importance for tumor control is evident and we will further expand on this aspect in future in vivo studies. In line with our findings, induction of T cell proliferation and activation in vitro has also been described after conventional PDT [26,31,42].

One important aspect to highlight is that although NB-PDT induces a large extent of necrosis, the immunogenicity here observed is not purely a passive effect of accidental necrosis and subsequent cell content release. It has been shown that cell lysates obtained by the freeze-thaw method, mimicking accidental necrosis, often fail to induce a marked upregulation of surface maturation markers on DCs and to activate T cells, while inducing very high levels of secreted IL-10 by DCs [26,31,34]. This supports the fact that NB-PDT leads to tumor cell death with a distinct immunogenic profile than mere accidental necrosis. Although the exact mechanism of how a PS localized on the cell membrane leads to ICD is still not fully elucidated, some clues have been given for antibody-targeted PDT, which can likely also be valid for NB-PDT. It has been shown that antibody-targeted PDT induces tiny perforations on the plasma membrane, followed by ionic imbalance and increase in membrane permeability, leading to cell rupture, necrosis and ICD [43]. In the same line, it has also been reported that the anticancer peptide RT53 induces a non-regulated form of necrosis which is immunogenic, including disruption of plasma membrane, release of intracellular content, and chaperone exposure via a distinct mechanism which does not follow the “canonical” pathways elicited by other ICD inducers [44]. In these cases, such as with NB-PDT, ICD is suggested to begin with cell membrane damage, possibly leading to ionic imbalance and the surface exposure of chaperones, which has been suggested to be controlled by endoplasmic reticulum Ca^2+^ levels [45], though additional studies should explore this further.

Altogether, our in vitro study shows that the presence of ICD markers and activation of immune cells induced by NB-PDT is more robust and pronounced when using a tumor cell line with high expression levels of the target. We expect that NB-PDT on tumor cells with moderate target expression will lead to more pronounced effects in vivo than in vitro, where tumor vasculature damage also plays a role. It has already been demonstrated that NB-PDT induces extensive tumor damage on a model with moderate expression levels [7] and, in addition, antibody-targeted PDT has been reported to trigger antitumor immunity against tumor cells with moderate levels of target [46]. Another aspect to underline from our study is the moderate immunogenicity induced by the mild NB-PDT condition. Others have used the treated tumor cells themselves as the initial stimulant [26,30,31,34,39], while we are the first to rely on the immunomodulatory effects of the conditioned medium of the treated cells, without the use of immunoadjuvants [47]. In our setup, the importance of released immunogenic factors plays a more essential role, which is known to abundantly happen in cells dying by necrosis (highly cytotoxic NB-PDT) in comparison to (immunogenic) apoptotic cells. The mild NB-PDT might rely on a combination of released and exposed molecules on the membrane of dying cells and a co-incubation setup may yield more promising results. Others have focused their efforts on using PDT doses inducing ~90% of cell death for the stimulation of DCs, often with the goal of developing antitumor vaccines, thus comparison is difficult in this context. Interestingly, suboptimal PDT protocols in vivo are potent immunostimulants despite the initial low tumor damage [48], thus it seems reasonable to believe that NB-PDT doses resulting in lower cytotoxicity can also lead to immunostimulation in the in vivo setting.

In conclusion, we have shown that NB-PDT holds the potential to stimulate the immune system. This can make an initial local treatment, which causes extensive damage to the primary tumor, also a systemic treatment capable of fighting metastases and preventing recurrences. The initial steps reported here pave the way for a series of events which are deemed of paramount importance if NB-PDT is to be further developed towards the clinic. Our next efforts will be directed towards investigating the immune effects of NB-PDT in preclinical models, to determine the extent of the systemic effects induced and to assess the development of an antitumor immunity.

## 4. Materials and Methods

### 4.1. Cell Lines and Nanobody-Photosensitizer Conjugates

The human epidermoid carcinoma cell line A431 was purchased from ATCC, and the human head and neck carcinoma cell line scc-U8 was kindly provided by Dr. Robinson (Erasmus MC, Rotterdam, The Netherlands). A431 cells are used as a reference cell line which overexpresses EGFR (0.5–3.5 × 10^6^ receptors per cell), while scc-U8 cells are a more representative tumor cell line with 36% EGFR expression relative to A431 cells [19,49]. Both cell lines were cultured in Dulbecco’s Modified Eagle’s Medium (DMEM) (Lonza, Basel, Switzerland) supplemented with 10% foetal bovine serum (FBS) (Sigma-Aldrich, Zwijndrecht, The Netherlands), 100 U/mL penicillin and 100 µg/mL streptomycin (Sigma-Aldrich). Cells were cultured at 37 °C and 5% CO_2_.

The EGFR-targeting monomeric NB 7D12 and the biparatopic NB 7D12-9G8, as well as their conjugation to the PS IRDye700DX N-hydroxysuccinimidine ester (LI-COR, Biosciences, Lincoln, NE, USA, cat no. 929-70011), have been previously described [7].

### 4.2. Nanobody-Targeted Photodynamic Therapy

A431 or scc-U8 cells were seeded in 96-well plates (10.000 and 15.000 cells/well, respectively) one day before the assay. The next day, cells were washed once with PDT medium, i.e., DMEM without phenol red (Lonza) supplemented with 10% FBS and antibiotics, and the concentration of conjugate to achieve 50% (LD50) or 100% (LD100) of cell death was added (1 and 25 nM for A431 cells, 10 and 100 nM for scc-U8 cells) (see Figure S1). Cells were incubated with the conjugates for 30 min at 37 °C. Thereafter, cells were washed twice with PDT medium and illuminated with 5 mW/cm^2^ for 33 min in the case of A431 cells, or 7 mW/cm^2^ for 59 min in the case of scc-U8 cells (equal to 10 and 25 J/cm^2^, respectively). Fluence rate was measured with an Orion/PD optometer (Ophir Optronics, Jerusalem, Israel) and light applied using a 690 nm laser (Modulight ML7700, Tampere, Finland). Cells were always seeded in two separate 96-well plates: one plate receiving light including conditions without conjugate (only light control), and LD50 and LD100 concentrations (NB-PDT); and another plate not illuminated and featuring the controls of LD100 (only conjugate control) and untreated cells. 

### 4.3. Mechanism of Cell Death

Right after NB-PDT, CellEvent caspase 3/7 green detection reagent (caspase 3/7) (Invitrogen, Carlsbad, CA, USA, cat no. R37111) and propidium iodide (PI) (Invitrogen, P5366) were added to the cells in a final dilution of 1:10 and 1:1000, respectively. Well plates were placed back in the incubator for 2 h and, hereafter, cells were imaged with an EVOS microscope (Thermo Fisher Scientific, Perbio Science Nederland, Etten-Leur, The Netherlands) using transmitted light, the GFP light cube for caspase 3/7, and the RFP light cube for PI (20x objective). Plates were returned to the incubator and imaged again 18 h after NB-PDT.

### 4.4. Cellular Localization of HSP70 and HMGB1 on Tumor Cells

NB-PDT was performed as described above, with the only difference being that cells were seeded on 16 wells Lab-Tek Chamber Slides (Thermo Fisher Scientific, 178599). For HSP70 detection, chamber slides were returned to the incubator after NB-PDT for 4 h, followed by 10 min at room temperature and 10 min at 4 °C. Cells were washed with medium (DMEM without phenol red supplemented with 25 mM HEPES and 2% BSA, pH 7.2) and the primary antibody Mouse anti-HSP70 (Thermo Fisher Scientific, MA3-009) was added for 1 h at 4 °C (1:100 in medium). Cells were fixed with 4% PFA (Merck, Haarlem, The Netherlands) for 15 min at room temperature and the secondary antibody Goat anti-Mouse Alexa555 (Molecular probes, A21424) was added for 1 h at room temperature (1:200 in PBS + 2% BSA). Cells were stained with DAPI (Roche, Basel, Switzerland) and imaged with a Confocal Laser Scanning Microscope (Carl Zeiss Microscopy GmbH, Germany, LSM700) using a plan-apochromat 63x/1.40 Oil DIC objective. 

For HMGB1 detection, chamber slides were returned to the incubator after NB-PDT for 4 h, followed by 10 min at room temperature. Cells were fixed with 4% PFA and permeabilized with 0.2% Triton X-100 (Sigma-Aldrich) for 15 min at room temperature. The primary antibody Rabbit anti- HMGB1 (Invitrogen, PA1-16926) was added for 1 h at room temperature (1:50 in in PBS + 2% BSA), followed by staining with the secondary antibody Donkey anti-Rabbit Alexa488 (Invitrogen, A21206) for 1 h at room temperature (1:500 in PBS + 2% BSA). 

### 4.5. Detection of ATP in the Supernatant of Tumor Cells

NB-PDT was performed, plates were placed back in the incubator and supernatants were collected 4 h later. Cell debris was removed by spinning down, supernatants were added to a white 96-well plate (Greiner Bio-One, Alphen a/d Rijn, The Netherlands, cat no. 655075) and mixed with CellTiter-Glo 3D (Promega, Madison, WI, USA, cat no. G9681) in a 1:1 ratio. Plates were incubated for 5 min on a horizontal orbital microplate shaker followed by an incubation of 25 min at room temperature, protected from light. Luminescence was measured with a GloMax 96™ microplate luminometer (Promega). Assay performance was verified each time by including ATP standards (Promega, P1132).

### 4.6. Detection of HSP70 in the Supernatant of Tumor Cells

NB-PDT was performed as described above, plates were placed back in the incubator and the supernatants were collected 24 h later. Cell debris was removed and a Human HSP70 ELISA kit (Invitrogen, BMS2087) was used with the supernatants, according to the manufacturer’s protocol. 

### 4.7. Detection of IL-1β, IL-6, and IL-8 as Well as in the Supernatant of Tumor Cells

A431 cells or scc-U8 cells were seeded on 6 cm petri dishes (650.000 and 700.000 cells/dish, respectively). The next day, NB-PDT was performed as described above and, afterwards, dishes were placed back in the incubator for 24 h. Supernatants were collected and concentrated using 3K Amicon Ultra Centrifugal Filters (Merck, UFC900324). IL-6, IL-8, and IL-1β were then detected in the concentrated supernatants using a Human Magnetic Luminex Assay (R&D Systems, Minneapolis, MN, USA), following the protocol of the manufacturer.

### 4.8. Generation, Stimulation and Analysis of Dendritic Cells Maturation

Human peripheral blood mononuclear cells (PBMCs) were isolated from buffy coats of healthy donors by means of density gradient centrifugation using LeucoSep tubes (Greiner Bio-One), followed by CD14+ monocyte isolation using magnetic negative selection with a Monocyte Isolation Kit (Miltenyi Biotec, Bergisch Gladbach, Germany, cat no. 130-117-337). Monocytes were cultured for 7 days in Roswell Park Memorial Institute (RPMI) 1640 with L-glutamine (Thermo Fisher Scientific) supplemented with 10% fetal calf serum (Bodinco, Alkmaar, The Netherlands), 100 U/mL penicillin/streptomycin (Thermo Fisher Scientific), 100 ng/mL IL-4 (ProSpec, Ness-Ziona, Israel), and 60 ng/mL GM-CSF (ProSpec). Immature moDCs were harvested at day 8 and seeded into a 96-well plate at a density of 1 × 10^5^ cells/well in culture medium without IL-4 and GM-CSF. Immature moDCs were either left unstimulated, stimulated with 100 ng/mL LPS (InvivoGen, San Diego, CA, USA, cat no. *E. coli* 055:B5) or with tumor conditioned media from different NB-PDT conditions. On average, 1 × 10^5^ moDCs were incubated with supernatant of 3 × 10^4^ tumor cells. After 24 h of stimulation, moDCs were collected, stained for surface markers and analyzed by flow cytometry using a FACS Canto II (BD Biosciences, San Jose, CA, USA). Cells were stained with antibodies against the following markers: CD11c (PerCP- e710), CD14 (APC), CD86 (PE-Cy7) and MHCII (PE) (eBioscience, San Diego, CA, USA), as well as the viability dye YO-PRO1 (Thermo Fisher Scientific). Expression of CD86 on moDCs was quantified as % of positive cells, due to its bimodal distribution, while mean fluorescence intensity (MFI) was used to denote MHCII expression. Furthermore, supernatants were collected and stored at −20 °C to later on measure IL- 12p40, IL-1β and IL-10 levels by Luminex (R&D Systems), according to the manufacturer’s protocols. 

### 4.9. Generation and Analysis of CD4+ T Cells Activation

Human PBMCs were isolated from buffy coats of healthy donors by means of density gradient centrifugation using LeucoSep tubes (Greiner), followed by CD4+ cells isolation using magnetic negative selection with a Naïve CD4+ T cell isolation kit II human (Miltenyi Biotec, 130-094-131). Naïve CD4+ cells were labeled with the proliferation dye CFSE (Thermo Fisher Scientific, C34570) and co- cultured with allogeneic moDCs (stimulated as described above) at DC:T cell ratio of 1:10 in Iscove’s Modified Dulbecco’s Medium (IMDM) with glutamax (Gibco, Invitrogen, United Kingdom, cat no. 31980-22), supplemented with 10% fetal calf serum, 100 U/mL penicillin/streptomycin, 20 µg/mL apo-transferrine and 50 µM β-mercaptoethanol. After 6 days of co-incubation, cells were collected and stained with antibodies anti-CD4-PerCP-Cy5.5 (eBioscience, 45-0048-42) and anti-CD3-APC (BioLegend, San Diego, CA, USA, cat no. 300412). Samples were analyzed by flow cytometry using a FACS Canto II (BD Biosciences) and the proliferating fraction identified based on the CFSE-negative population from samples with CFSE-labeled CD4+ T cells alone. Furthermore, the supernatants derived from the co-incubation were collected and stored at −20 °C to measure later IFNγ levels by ELISA (Invitrogen, 88-7316-22), according to the manufacturer’s protocol. 

### 4.10. Statistical Analysis

Data were analyzed with GraphPad Prism 8 software using one-way ANOVA with a Tukey post-hoc test to compare between conditions. Statistical significance was displayed as * *p* ≤ 0.05, ** *p* ≤ 0.01, and *** *p* ≤ 0.001.

## 5. Conclusions

Our results show, for the first time, that NB-PDT induces rapid necrosis leading to the exposure and release of DAMPs and cytokines from dying tumor cells. Consequently, maturation of DCs and T cell activation is induced. These findings suggest that NB-PDT, like conventional PDT, can trigger ICD and potentially stimulate the immune system, providing systemic effects to a selective and local therapy.

## Supplementary Materials

The following are available online at https://www.mdpi.com/2072-6694/12/4/978/s1, Figure S1: Cytotoxicity induced by NB-PDT on different cell lines, Figure S2: NB-PDT controls on A431 cells for different assays, Figure S3: Effect of NB-PDT with the biparatopic conjugate 7D12-9G8-PS on tumor cells, Figure S4: Phenotypic maturation of moDCs incubated with supernatant of NB-PDT treated scc-U8 cells, Figure S5: Tumor supernatant after NB-PDT presents TLR4 agonistic activity.

## References

[1] Van Straten D., Mashayekhi V., de Bruijn H.S., Oliveira S., Robinson D.J. (2017). Oncologic photodynamic therapy: Basic principles, current clinical status and future directions. Cancers.

[2] Dolmans D.E.J.G.J., Fukumura D., Jain R.K. (2003). Photodynamic therapy for cancer. Nat. Rev. Cancer.

[3] Castano A.P., Mroz P., Hamblin M.R. (2006). Photodynamic therapy and anti-tumour immunity. Nat. Rev. Cancer.

[4] Beltrán Hernández I., Yu Y., Ossendorp F., Korbelik M., Oliveira S. (2020). Preclinical and Clinical Evidence of Immune Responses Triggered in Oncologic Photodynamic Therapy: Clinical Recommendations. J. Clin. Med..

[5] Agostinis P., Berg K., Cengel K., Foster T., Girotti A., Gollnick S., Hahn S., Hamblin M., Juzeniene A., Kessel D. (2011). Photodynamic Therapy of cancer: An update. CA Cancer J. Clin..

[6] Cognetti D., Curry J.M., Gillenwater A.M., William W.N., Kochuparambil S.T., McDonald D., Fidler M., Stenson K.M., Vasan N.R., Razaq M.A. (2018). A Phase 2a, Multicenter, Open-Label Study of RM-1929 Photoimmunotherapy in Patients With Recurrent Head And Neck Cancer. Int. J. Radiat. Oncol..

[7] Van Driel P.B.A.A., Boonstra M.C., Slooter M.D., Heukers R., Stammes M.A., Snoeks T.J.A., De Bruijn H.S., Van Diest P.J., Vahrmeijer A.L., Van Bergen En Henegouwen P.M.P. (2016). EGFR targeted nanobody-photosensitizer conjugates for photodynamic therapy in a pre-clinical model of head and neck cancer. J. Control. Release.

[8] Heukers R., van Bergen en Henegouwen P.M.P., Oliveira S. (2014). Nanobody–photosensitizer conjugates for targeted photodynamic therapy. Nanomedicine Nanotechnol. Biol. Med..

[9] Oliveira S., Heukers R., Sornkom J., Kok R.J., van Bergen En Henegouwen P.M.P. (2013). Targeting tumors with nanobodies for cancer imaging and therapy. J. Control. Release.

[10] Oliveira S., Van Dongen G.A.M.S., Stigter-Van Walsum M., Roovers R.C., Stam J.C., Mali W., Van Diest P.J., Van Bergen En Henegouwen P.M.P. (2012). Rapid visualization of human tumor xenografts through optical imaging with a near-infrared fluorescent anti-epidermal growth factor receptor nanobody. Mol. Imaging.

[11] Beltrán Hernández I., Rompen R., Rossin R., Xenaki K.T., Katrukha E.A., Nicolay K., Van Bergen P., Grüll H., Oliveira S. (2019). Imaging of Tumor Spheroids, Dual-Isotope SPECT, and Autoradiographic Analysis to Assess the Tumor Uptake and Distribution of Different Nanobodies. Mol. Imaging Biol..

[12] Deken M.M., Kijanka M.M., Beltrán Hernández I., Slooter M.D., van Diest P.J., de Bruijn H.S., van Bergen en Henegouwen P.M., Lowik C.W., Robinson D.J., Vahrmeijer A.L. (2020). Nanobody-targeted photodynamic therapy induces significant tumor regression of trastuzumab-resistant HER2-positive breast cancer, after a single treatment session. J. Control. Release.

[13] Heukers R., Mashayekhi V., Ramirez-Escudero M., de Haard H., Verrips T.C., van Bergen en Henegouwen P.M.P., Oliveira S. (2019). VHH-Photosensitizer Conjugates for Targeted Photodynamic Therapy of Met-Overexpressing Tumor Cells. Antibodies.

[14] De Groof T.W.M., Mashayekhi V., Shu Fan T., Bergkamp N.D., Sastre Torano J., Van Senten J.R., Heukers R., Smit M.J., Oliveira S. (2019). Nanobody-Targeted Photodynamic Therapy Selectively Kills Viral GPCR-Expressing Glioblastoma Cells. Mol. Pharm..

[15] De Bruijn H.S., Mashayekhi V., Schreurs T.J.L., van Driel P.B.A.A., Strijkers G.J., van Diest P.J., Lowik C.W.G.M., Seynhaeve A.L.B., ten Hagen T.L.M., Prompers J.J. (2020). Acute cellular and vascular responses to photodynamic therapy using EGFR-targeted nanobody-photosensitizer conjugates studied with intravital optical imaging and magnetic resonance imaging. Theranostics.

[16] Garg A.D., Agostinis P. (2014). ER stress, autophagy and immunogenic cell death in photodynamic therapy-induced anti-cancer immune responses. Photochem. Photobiol. Sci..

[17] Garg A.D., Galluzzi L., Apetoh L., Baert T., Zitvogel L., Agostinis P. (2015). Molecular and translational Classifications of DaMPs in immunogenic Cell Death. Front. Immunol..

[18] Maeding N., Verwanger T., Krammer B. (2016). Boosting Tumor-Specific Immunity Using PDT. Cancers.

[19] Peng W., de Bruijn H.S., Farrell E., Sioud M., Mashayekhi V., Oliveira S., van Dam G.M., Roodenburg J.L.N., Witjes M.J.H., Robinson D.J. (2018). Epidermal growth factor receptor (EGFR) density may not be the only determinant for the efficacy of EGFR-targeted photoimmunotherapy in human head and neck cancer cell lines. Lasers Surg. Med..

[20] Driehuis E., Spelier S., Beltrán Hernández I., De Bree R., Willems S.M., Clevers H., Oliveira S. (2019). Patient-Derived Head and Neck Cancer Organoids Recapitulate EGFR Expression Levels of Respective Tissues and Are Responsive to EGFR-Targeted Photodynamic Therapy. J. Clin. Med..

[21] Anzengruber F., Avci P., De Freitas L.F., Hamblin M.R. (2015). T-cell mediated anti-tumor immunity after photodynamic therapy: Why does it not always work and how can we improve it?. Photochem. Photobiol. Sci..

[22] Heukers R., Vermeulen J.F., Fereidouni F., Bader A.N., Voortman J., Roovers R.C., Gerritsen H.C., Van Bergen En Henegouwen P.M.P. (2013). Endocytosis of EGFR requires its kinase activity and N-terminal transmembrane dimerization motif. J. Cell Sci..

[23] Nicotera P., Leist M., Ferrando-May E. (1998). Intracellular ATP, a switch in the decision between apoptosis and necrosis. Toxicol. Lett..

[24] Thompson S.A., Aggarwal A., Singh S., Adam A.P., Tome J.P.C., Drain C.M. (2018). Compromising the plasma membrane as a secondary target in photodynamic therapy-induced necrosis. Bioorganic Med. Chem..

[25] Ogawa M., Tomita Y., Nakamura Y., Lee M., Lee S., Tomita S., Nagaya T., Sato K., Yamauchi T., Trepel B. (2017). Immunogenic cancer cell death selectively induced by near infrared photoimmunotherapy initiates host tumor immunity. Oncotarget.

[26] Ji J., Fan Z., Zhou F., Wang X., Shi L., Zhang H., Wang P., Yang D., Zhang L., Chen W.R. (2015). Improvement of DC vaccine with ALA-PDT induced immunogenic apoptotic cells for skin squamous cell carcinoma. Oncotarget.

[27] Garg A.D., Nowis D., Golab J., Vandenabeele P., Krysko D.V., Agostinis P. (2010). Immunogenic cell death, DAMPs and anticancer therapeutics: An emerging amalgamation. BBA Rev. Cancer.

[28] Panzarini E., Inguscio V., Fimia G.M., Dini L. (2014). Rose Bengal Acetate PhotoDynamic Therapy (RBAc-PDT) Induces Exposure and Release of Damage-Associated Molecular Patterns (DAMPs) in Human HeLa Cells. PLoS ONE.

[29] Korbelik M., Sun J., Cecic I. (2005). Photodynamic Therapy − Induced Cell Surface Expression and Release of Heat Shock Proteins: Relevance for Tumor Response. Cancer Res..

[30] Wang X., Ji J., Zhang H., Fan Z., Zhang L., Shi L., Zhou F., Chen W.R., Wang H., Wang X. (2015). Stimulation of dendritic cells by DAMPs in ALA-PDT treated SCC tumor cells. Oncotarget.

[31] Zheng Y., Yin G., Le V., Zhang A., Chen S., Liang X., Liu J. (2016). Photodynamic-therapy Activates Immune Response by disrupting Immunity Homeostasis of Tumor Cells, which Generates Vaccine for Cancer Therapy. Int. J. Biol. Sci..

[32] Tesniere A., Panaretakis T., Kepp O., Apetoh L., Ghiringhelli F., Zitvogel L., Kroemer G. (2008). Molecular characteristics of immunogenic cancer cell death. Cell Death Differ..

[33] Tanaka M., Kataoka H., Yano S., Sawada T. (2016). Immunogenic cell death due to a new photodynamic therapy (PDT) with glycoconjugated chlorin (G-chlorin). Oncotarget.

[34] Garg A.D., Krysko D.V., Verfaillie T., Kaczmarek A., Ferreira G.B., Marysael T., Rubio N., Firczuk M., Mathieu C., Roebroek A.J.M. (2012). A novel pathway combining calreticulin exposure and ATP secretion in immunogenic cancer cell death. EMBO J..

[35] Waugh D.J.J., Wilson C. (2008). The Interleukin-8 Pathway in Cancer. Clin. Cancer Res..

[36] Kawczyk-Krupka A., Czuba Z., Latos W., Wasilewska K., Verwanger T., Krammer B., Siero A. (2018). Influence of ALA-mediated photodynamic therapy on secretion of interleukins 6, 8 and 10 by colon cancer cells in vitro. Photodiagnosis Photodyn. Ther..

[37] Usuda J., Okunaka T., Furukawa K., Tsuchida T., Kuroiwa Y., Ohe Y., Saijo N., Nishio K., Konaka C., Kato H. (2001). Increased cytotoxic effects of photodynamic therapy in IL-6 gene transfected cells via enhanced apoptosis. Int. J. Cancer.

[38] Koon H., Lo K., Leung K., Lung M.L., Chang C.C., Wong R.N., Leung W., Mak N. (2010). Photodynamic therapy-mediated modulation of inflammatory cytokine production by Epstein—Barr virus-infected nasopharyngeal carcinoma cells. Cell. Mol. Immunol..

[39] Gollnick S.O., Vaughan L., Henderson B.W. (2002). Generation of Effective Antitumor Vaccines Using Photodynamic Therapy. Cancer Res..

[40] Schoenborn J.R., Wilson C.B. (2007). Regulation of Interferon- g During Innate and Adaptive Immune Responses. Adv. Immunol..

[41] Galaine J., Borg C., Godet Y., Adotévi O. (2015). Interest of Tumor-Specific CD4 T Helper 1 Cells for Therapeutic Anticancer Vaccine. Vaccines.

[42] Garg A.D., Dudek A.M., Ferreira G.B., Verfaillie T., Vandenabeele P., Krysko D.V., Mathieu C., Agostinis P., Garg A.D., Dudek A.M. (2013). ROS-induced autophagy in cancer cells assists in evasion from determinants of immunogenic cell death. Autophagy.

[43] Nakajima K., Takakura H., Shimizu Y., Ogawa M. (2018). Changes in plasma membrane damage inducing cell death after treatment with near-infrared photoimmunotherapy. Cancer Sci..

[44] Pasquereau-kotula E., Habault J., Kroemer G., Poyet J.-L. (2018). The anticancer peptide RT53 induces immunogenic cell death. PLoS ONE.

[45] Tufi R., Panaretakis T., Bianchi K., Criollo A., Fazi B., Di Sano F., Tesniere A., Kepp O., Paterlini-Brechot P., Zitvogel L. (2008). Reduction of endoplasmic reticulum Ca2+ levels favors plasma membrane surface exposure of calreticulin. Cell Death Differ..

[46] Nagaya T., Friedman J., Maruoka Y., Ogata F., Okuyama S., Clavijo P.E., Choyke P.L., Allen C., Kobayashi H. (2019). Host immunity following near-infrared photoimmunotherapy is enhanced with PD-1 checkpoint blockade to eradicate established antigenic tumors. Cancer Immunol. Res..

[47] Xia Y., Gupta G.K., Castano A.P., Mroz P., Avci P., Hamblin M.R. (2014). CpG oligodeoxynucleotide as immune adjuvant enhances photodynamic therapy response in murine metastatic breast cancer. J. Biophotonics.

[48] Henderson B.W., Gollnick S.O., Snyder J.W., Busch T.M., Kousis P.C., Cheney R.T., Morgan J. (2004). Choice of Oxygen-Conserving Treatment Regimen Determines the Inflammatory Response and Outcome of Photodynamic Therapy of Tumors. Cancer Res..

[49] Haigler H., Asht J.F., Singert S.J., Cohen S. (1978). Visualization by fluorescence of the binding and internalization of epidermal growth factor in human carcinoma cells A-431. Proc. Natl. Acad. Sci. USA.

