# Supplementary material for: The Potential of Nanobody-Targeted Photodynamic Therapy to Trigger Immune Responses
Source: Cancers (Basel). 2020 Apr 15;12(4):978. doi: 10.3390/cancers12040978 (PMC7226123; doi:10.3390/cancers12040978)
Supplement: Supplementary file 1 [file cancers-12-00978-s001.pdf]

The potential of nanobody-targeted photodynamic therapy to trigger immune responses

Irati Beltrán Hernández [1], Mathieu Angelier [2], Tommaso Del Buono D'Ondes [1], Alessia Di Maggio [2], Yingxin Yu [2] and Sabrina Oliveira [1,2,*]

[1] Pharmaceutics, Department of Pharmaceutical Sciences, Faculty of Science, Utrecht University, 3584 CG Utrecht, The Netherlands

[2] Cell Biology, Neurobiology and Biophysics, Department of Biology, Faculty of Science, Utrecht University, 3584 CH Utrecht, The Netherlands

* Correspondence: s.oliveira@uu.nl; Tel.: +31 634103460

Supplementary Data

(a)

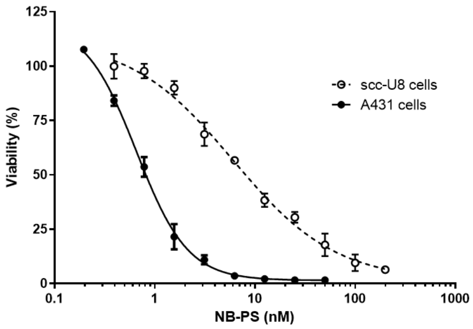

(b)

Cell line	LD50 (nM)
A431	0.86 ± 0.08
SCC-U8	9.17 ± 1.61

(c)

Cell line	LD50 (nM)	LD100 (nM)
A431	1	25
SCC-U8	10	100

Figure S1. Cytotoxicity induced by NB-PDT on different cell lines. A431 and scc-U8 cells were treated with NB-PDT with a concentration range of 7D12-PS conjugate. Cells were placed back in the incubator and cell viability was assessed 24 hours after the treatment using AlamarBlue reagent. (**a**), Cell viability curves (% relative to untreated cells) from which LD50 values were calculated. (**b**), Overview of the LD50 values for each cell line. Differences in the cytotoxicity of the treatment on the different cell lines are explained by the 3-fold higher EGFR levels on A431 cells. (**c**), Overview of the concentrations to achieve LD50 and LD100 used in this study for each cell line.

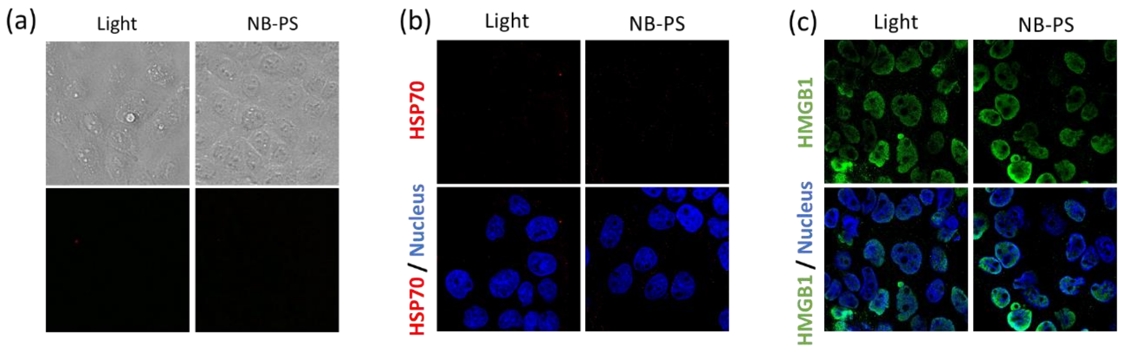

Figure S2. NB-PDT controls on A431 cells for different assays. (**a**), Tumor cells were subjected to either only light or only 7D12-PS conjugate (LD100) and stained with PI for necrotic cells and caspase 3/7 for apoptotic cells. Microscopy images were taken 18 hours later. Top panels depict the transmitted light image, while merged images of necrotic and apoptotic cells are shown on the bottom panels. (**b**) and (**c**) Tumor cells were subjected to the single components of PDT. Four hours later, either (**b**) extracellular staining of HSP70 (red) or (**c**) intracellular staining of HMGB1 (green) was performed. Cell nuclei were additionally stained with DAPI (blue). Top panels depict only the DAMP signal, while merged images are shown on the bottom panels. The same controls reported in this figure were also performed with scc-U8 cells, resulting in similar images.

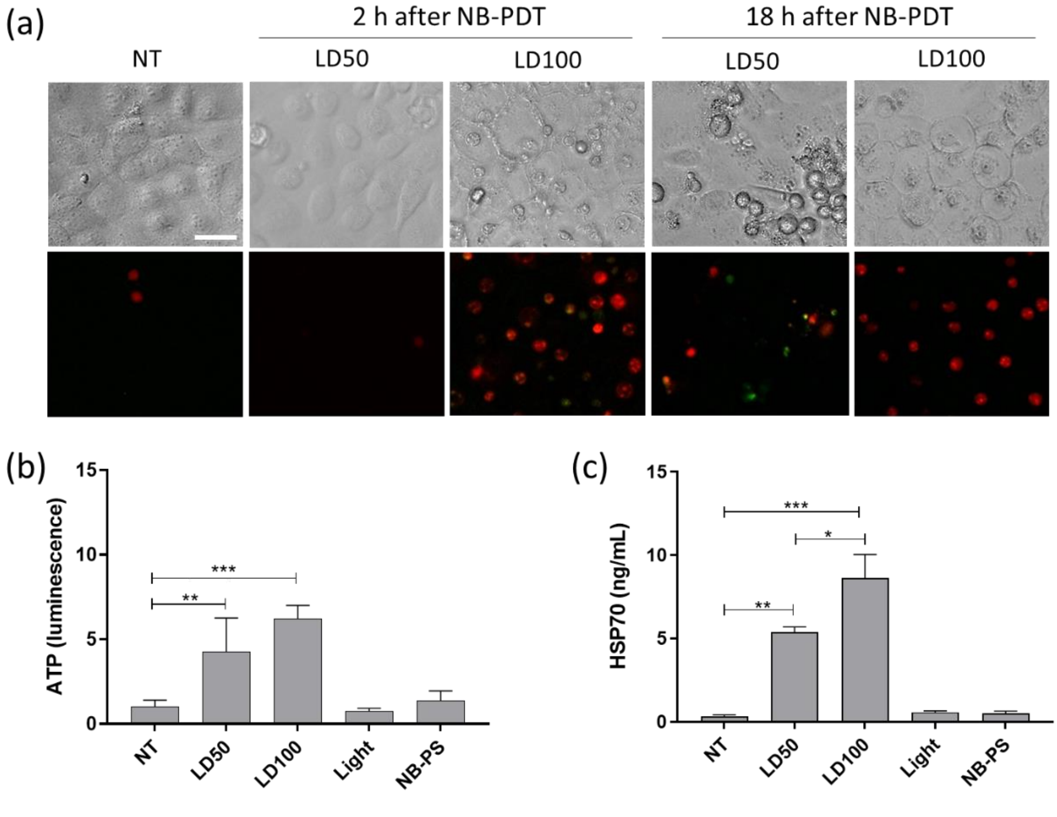

Figure S3. Effect of NB-PDT with the biparatopic conjugate 7D12-9G8-PS on tumor cells. (**a**), A431 cells were left untreated (NT) or treated with NB-PDT (LD50 or LD100) and stained with PI for necrotic cells (red) and caspase 3/7 for apoptotic cells (green). Microscopy images were taken 2 and 18 hours after the treatment. Top panels depict the transmitted light image, while merged images of necrotic and apoptotic cells are shown on the bottom

panels. Cells that are stained with both dyes appear orange and represent apoptotic cells at a later stage. Scale bar, 20µm. (**b**) and (**c**), A431 cells were left untreated or treated with NB-PDT (or controls consisting of only light or NB-PS conjugate). ATP in the supernatant was detected 4 hours later via a luminescence assay and graphs show luminescence values relative to untreated cells (b). Additionally, HSP70 in the supernatant was detected 24 hours after treatment using ELISA (c). Significance is displayed as *p ≤ 0.05, **p ≤ 0.01 and ***p ≤ 0.001.

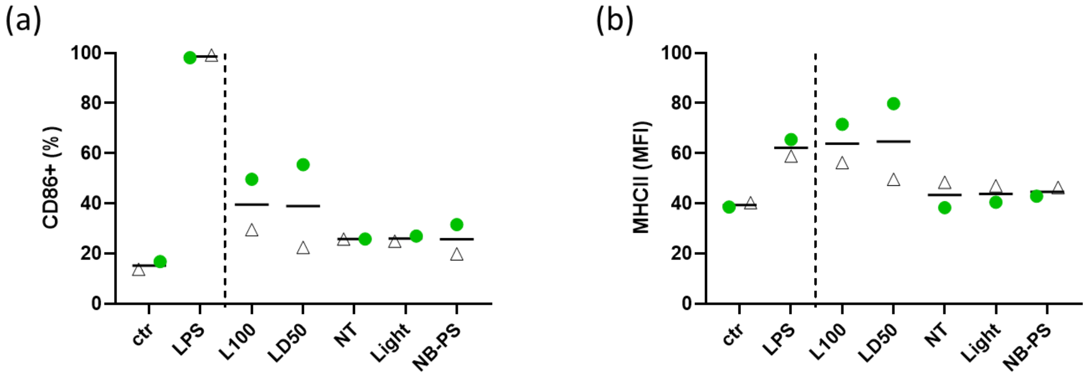

Figure S4. Phenotypic maturation of moDCs incubated with supernatant of NB-PDT treated scc-U8 cells. scc-U8 cells were left untreated (NT) or treated with NB-PDT using 7D12-PS (LD50 or LD100) and the respective controls (only light or NB-PS conjugate). The supernatant was collected 24 hours later and incubated with immature moDCs for another 24 hours. Surface marker expression on moDCs was measured with flow cytometry. A positive control was obtained by incubating moDCs with 100 ng/ml LPS for 24 hours (LPS), and a control consisting of unstimulated moDCs (ctr) was included. (**a**), Percentage of CD86 positive moDCs. (**b**), Median fluorescence intensity (MFI) corresponding to MHC II expression on moDCs. Each donor is represented by a different symbol (n=2).

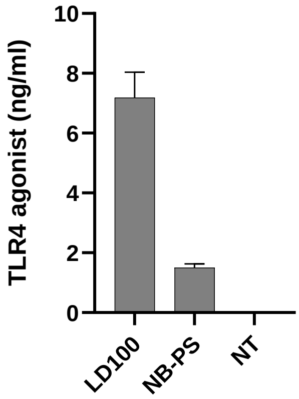

Figure S5. Tumor supernatant after NB-PDT presents TLR4 agonistic activity. A431 cells were left untreated (NT), treated with NB-PDT using 7D12-PS (LD100) or a control with only conjugate (NB-PS). The supernatant was collected 24 hours later and used in a reporter assay with the cell line HEK-Blue-hTLR4 cells (InvivoGen, hkb-htlr4) following the manufacturer's protocol. Tumor supernatant after treatment with highly cytotoxic NB-PDT showed TLR4 agonistic activity equivalent to 7.2 ng/ml of LPS.
